# Protective Effect of Ergothioneine against 7-Ketocholesterol-Induced Mitochondrial Damage in hCMEC/D3 Human Brain Endothelial Cells

**DOI:** 10.3390/ijms24065498

**Published:** 2023-03-13

**Authors:** Damien Meng-Kiat Leow, Irwin Kee-Mun Cheah, Zachary Wei-Jie Fong, Barry Halliwell, Wei-Yi Ong

**Affiliations:** 1 Department of Anatomy, Yong Loo Lin School of Medicine, National University of Singapore, Singapore 117594, Singapore; 2 Neurobiology Research Programme, Life Sciences Institute, National University of Singapore, Singapore 117456, Singapore; 3 Department of Biochemistry, Yong Loo Lin School of Medicine, National University of Singapore, Singapore 117596, Singapore

**Keywords:** ergothioneine, 7-ketocholesterol, oxysterols, cytoprotection

## Abstract

Recent findings have suggested that the natural compound ergothioneine (ET), which is synthesised by certain fungi and bacteria, has considerable cytoprotective potential. We previously demonstrated the anti-inflammatory effects of ET on 7-ketocholesterol (7KC)-induced endothelial injury in human blood-brain barrier endothelial cells (hCMEC/D3). 7KC is an oxidised form of cholesterol present in atheromatous plaques and the sera of patients with hypercholesterolaemia and diabetes mellitus. The aim of this study was to elucidate the protective effect of ET on 7KC-induced mitochondrial damage. Exposure of human brain endothelial cells to 7KC led to a loss of cell viability, together with an increase in intracellular free calcium levels, increased cellular and mitochondrial reactive oxygen species, a decrease in mitochondrial membrane potential, reductions in ATP levels, and increases in mRNA expression of *TFAM*, *Nrf2*, *IL-1β*, *IL-6* and *IL-8.* These effects were significantly decreased by ET. Protective effects of ET were diminished when endothelial cells were coincubated with verapamil hydrochloride (VHCL), a nonspecific inhibitor of the ET transporter OCTN1 (*SLC22A4*). This outcome demonstrates that ET-mediated protection against 7KC-induced mitochondrial damage occurred intracellularly and not through direct interaction with 7KC. OCTN1 mRNA expression itself was significantly increased in endothelial cells after 7KC treatment, consistent with the notion that stress and injury may increase ET uptake. Our results indicate that ET can protect against 7KC-induced mitochondrial injury in brain endothelial cells.

## 1. Introduction

There has been much recent interest in the roles of oxysterols in cardiovascular and neurodegenerative diseases. Auto-oxidation of cholesterol esters present in low-density lipoprotein (LDL) leads to the formation of oxidised LDL (Ox-LDL), along with its by-products. 7-ketocholesterol (7KC) is a major oxidation product of cholesterol found in human atherosclerotic plaque, and it was more atherogenic than cholesterol in some animal studies [1]. 7KC can be derived from the diet or endogenously produced [2]. It has been demonstrated to disrupt membrane fluidity (increased permeability) before inducing numerous stress response pathways and cell death [2]. 7KC is also involved in mediating the inflammatory mechanisms involved in progression of fibroatheroma plaques (reviewed in [3]). 7KC also induces inflammation in ARPE-19 retinal pigment epithelium cells through elevated expression of the cytokines VEGF, IL-6, and IL-8 via the AKT-PKCζ-NFκB, p38 MAPK, and ERK signalling pathways [4]. Increased brain 7KC levels have been found in rats after excitotoxic brain injury induced by kainic acid [5,6]. Moreover, 7KC induces metabolic dysfunction [2] and blood brain barrier (BBB) dysfunction [7,8]; 7KC levels are increased in patients with type 2 diabetes mellitus [9], Alzheimer’s disease [10] and Parkinson’s disease [11], compared to healthy controls. 7KC levels are also correlated with the number of concurrent coronary risk factors [12]. 7KC is cytotoxic to cells of the vascular wall, and it is capable of inducing apoptosis in endothelial cells and smooth muscle cells and necrosis in fibroblasts [13]. Moreover, 7KC can also induce an increase in intracellular free calcium, either through release from internal stores or influx via calcium channels, and can trigger neurotransmitter release in PC12 cells [14]. In addition, 7KC can injure intracellular organelles, such as the mitochondria, through alteration of membrane fluidity and build-up of oxidation products [2,15]. In addition to generating energy for cells, mitochondria also play a role in intracellular calcium buffering [16]. Mitochondrial dysfunction can be characterised by damage leading to excessive generation of reactive oxygen species (ROS), a decrease in mitochondrial membrane potential, cytochrome c release, ATP depletion and caspase 3 activation [17]. Dietary polyphenols, such as resveratrol, quercetin, and apigenin, have been shown to reduce 7KC-induced oxidative stress and apoptosis in Neuro 2A (N2a) cells [18].

Ergothioneine (ET) is a unique low-molecular weight dietary thiol/thione that can cross the blood-brain barrier. This compound accumulates at high levels in the body from the diet and may play important physiological roles in human health and development and possibly in the prevention and treatment of disease [19]. Blood levels of ET decline with age and with the onset of various diseases [20], especially neurodegenerative diseases [21]. ET was taken up by human brain microvascular endothelial cells (HBMECs) consistent with the presence of the ET transporter OCTN1 in these cells, and it protected the endothelial cells against oxidative stress induced by high glucose, pyrogallol, and xanthine oxidase plus xanthine [22]. In addition, ET had a protective effect on high glucose-induced oxidative stress in endothelial cells [23]. ET also reduced endothelial cell senescence linked to hyperglycaemia through the regulation of SIRT1 and SIRT6 signalling [24]. Furthermore, ET reduced the proinflammatory induction of adhesion molecule expression associated with atherogenesis in human aortic endothelial cells (HAECs) [25]. We previously demonstrated that ET is able to mitigate 7KC-induced inflammation in brain endothelial cells [26]. However, the mechanism of cellular protection in endothelial cells has yet to be elucidated. 7KC is known to cause toxicity through damage to mitochondria [15]; hence, we sought to determine whether preservation of cell viability by ET could be related to protection against 7KC-induced mitochondrial injury. Human brain endothelial cells were used as a model of oxysterol-induced BBB dysfunction and to investigate the potential cytoprotective mechanisms of ET—especially in relation to mitochondrial damage.

## 2. Results

### 2.1. Cell Death and Cell Viability Assay

**Figure 1:**
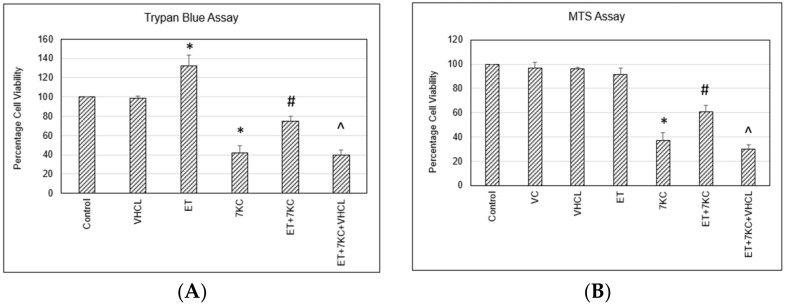
Cell death and cell viability assays. Treatment of cells with 30 μM 7KC resulted in death of brain endothelial cells using Trypan blue assay, and cell loss was significantly decreased by cotreatment with ET. ET alone increased cell viability using Trypan blue assay but not with MTS assay. (**A**) The protective effect of ET was abolished when the cells were treated with an inhibitor of OCTN1 and VHCL, together with ET. Comparable results were obtained using MTS assay. (**B**) ET was found to block 7KC-induced injury in this assay, and the effect was abolished when the cells were incubated with VHCL. VC: Vehicle control; ET: 1 mM ET; VHCL: 100 μM VHCL; 7KC: 30 μM 7KC. Data are represented as the mean ± SD (n = 4). * Significant difference compared to controls (*p* < 0.05). # Significant difference compared to 7KC. ^ Significant difference compared to ET + 7KC. Data were analysed by one-way ANOVA with Bonferroni’s multiple comparison post-hoc test.

Trypan blue assay relies on cellular membrane integrity, whereas MTS assay is based on intracellular metabolic activity. Treatment of cells with 30 μM 7KC resulted in approximately 60% death and loss of metabolic activity of brain endothelial cells visualised using the Trypan blue assay and MTS assay, respectively. Cell death was significantly reduced by cotreatment with ET (Figure 1). VHCL by itself did not have any effect on cell death. However, the protective effect of ET was abolished when cells were treated with an inhibitor of OCTN1 and VHCL, together with ET (Figure 1A). Comparable results were obtained using the MTS assay. ET was found to prevent 7KC-induced loss of metabolic activity; however, this effect was abolished when cells were pretreated with VHCL (Figure 1B).

### 2.2. OCTN1 Gene (SLC22A4) Expression and ET Uptake in Cells

**Figure 2:**
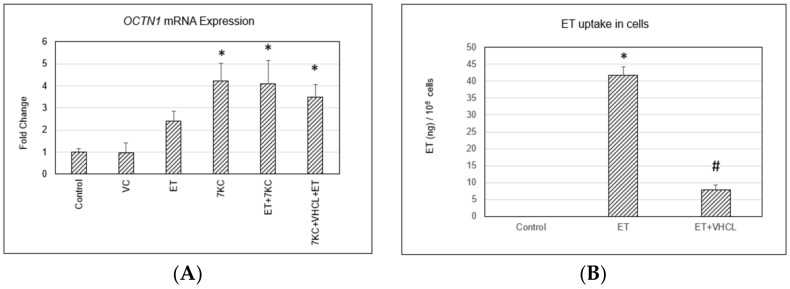
Quantitative RT-PCR of the *OCTN1* gene and ET uptake in cells. (**A**) Treatment of cells with VHCL did not alter the expression of *OCTN1*, and treatment with ET induced a non-significant, 2-fold increase in transporter expression. In contrast, 7KC induced a significant, 4-fold increase in *OCTN1* mRNA expression. This increase was not affected by the addition of ET or VHCL. (**B**) Treatment of cells with ET significantly increased ET levels in cells, but levels were significantly reduced by cotreatment with VHCL. VC: Vehicle control; ET: 1 mM ET; VHCL: 100 μM VHCL; 7KC: 30 μM 7KC. Data are represented as the mean ± SD (n = 3). * Significant difference compared to controls (*p* < 0.05). # Significant difference compared to ET. Data were analysed by one-way ANOVA with Bonferroni’s multiple comparison post-hoc test.

Treatment of cells with VHCL or ET did not significantly alter the expression of the *SLC22A4* gene encoding the ET transporter OCTN1. However, 7KC induced a significant, 4-fold increase in *OCTN1* mRNA expression in endothelial cells. This increase was unchanged by cotreatment with ET or VHCL (Figure 2A). ET was not detected in control cells, but intracellular ET levels significantly increased after treatment with ET. Addition of VHCL decreased ET uptake (Figure 2B).

### 2.3. Intracellular Free Calcium Levels

**Figure 3:**
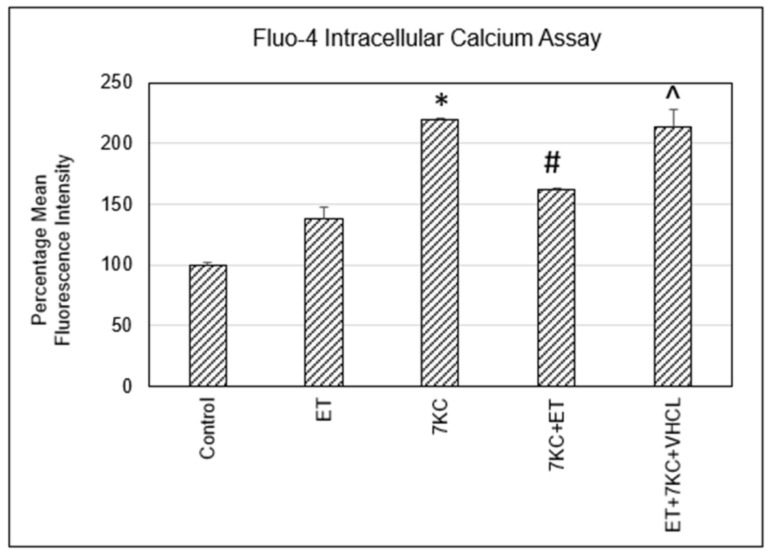
7KC induced an increase in intracellular free calcium as detected by Fluo-4 assay using flow cytometry. This increase was significantly modulated by ET. Addition of VHCL abrogated the increase in intracellular free calcium. ET: 1 mM ET; VHCL: 100 μM VHCL; 7KC: 30 μM 7KC. Data are represented as the mean ± SD (n = 6). * Significant difference compared to controls (*p* < 0.05). # Significant difference compared to 7KC. ^ Significant difference compared to ET + 7KC. Data were analysed by one-way ANOVA with Bonferroni’s multiple comparison post-hoc test.

7KC induced an increase in intracellular free calcium as detected by Fluo-4 assay using flow cytometry. Addition of ET significantly decreased the 7KC-induced increase in intracellular free calcium, but addition of VHCL inhibited this effect (Figure 3).

### 2.4. Overall ROS Production

**Figure 4:**
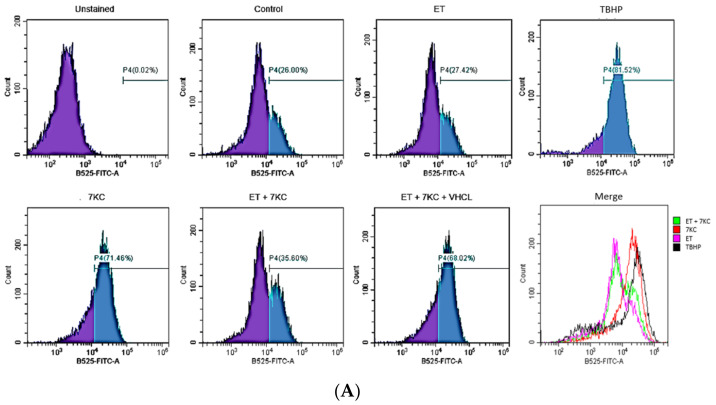

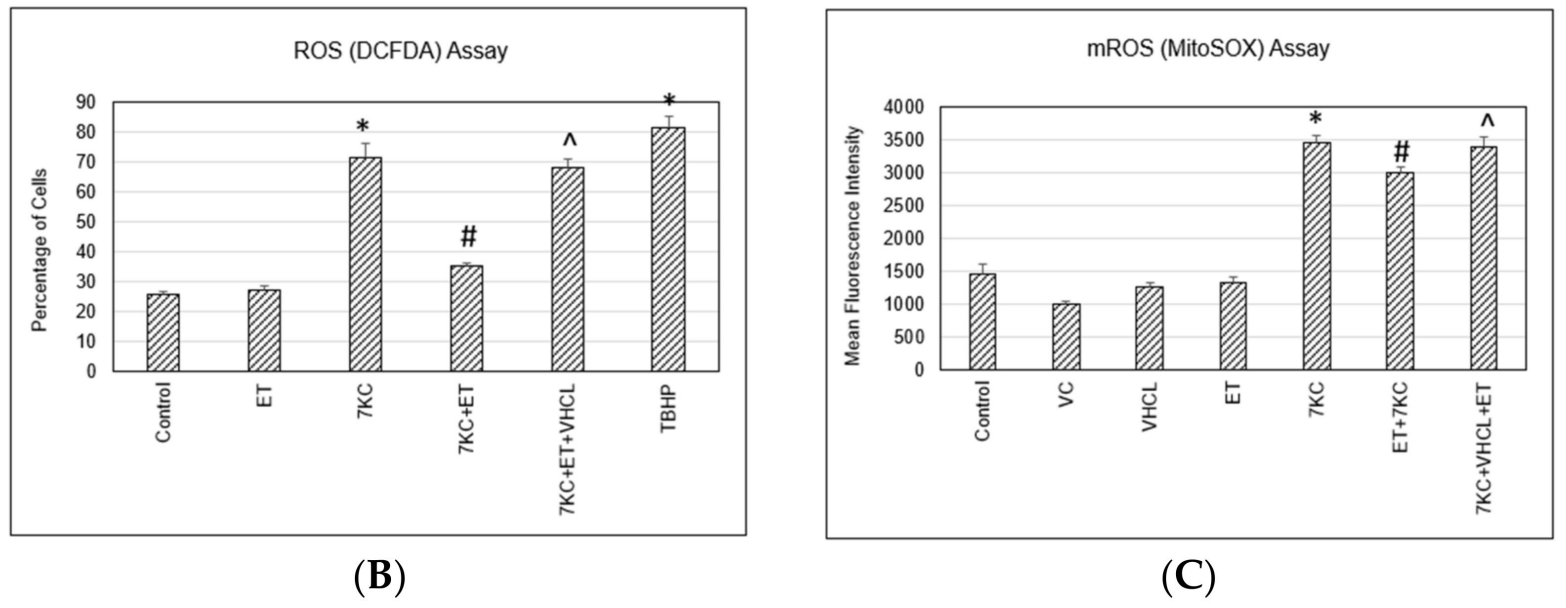
Overall ROS and mitochondrial ROS (mROS) production. (**A**) Individual histograms for overall ROS detection using DCFDA assay. Green: ET + 7KC; Red: 7KC only; Purple: ET only; Black: TBHP only. (**B**) 7KC appeared to induce an increase in overall ROS (ROS) production in cells, as shown by DCFDA assay. Addition of ET significantly decreased the ROS production, and such protection was abrogated by VHCL. (**C**) 7KC also induced an increase in mROS production in cells, as shown by MitoSOX assay, and this increase was similarly reduced by ET. VC: Vehicle control; ET: 1mM ET; VHCL: 100 μM VHCL; 7KC: 30 μM 7KC; TBHP: 50 μM TBHP. Data are represented as the mean ± SD (n = 3). * Significant difference compared to controls (*p* < 0.05). # Significant difference compared to 7KC. ^ Significant difference compared to ET + 7KC. Data were analysed by one-way ANOVA with Bonferroni’s multiple comparison post-hoc test.

7KC appeared to induce an increase in overall ROS production in cells, as shown by DCFDA assay, which of course has some problems with specificity [27]. Positive control TBHP also induced overall ROS production in cells. The addition of ET significantly reduced the DCFDA signal from approximately 70% to 35%; however, this effect was abrogated by VHCL tert-butyl hydroperoxide (TBHP), which is a positive control for the DCFDA assay (Figure 4A). To support the DCFDA assay results and verify mitochondrial ROS (mROS) production, MitoSOX assay was used. 7KC induced a significant increase in mROS production in cells, and this increase was reduced with ET (Figure 4B).

### 2.5. Mitochondrial Membrane Potential

**Figure 5:**
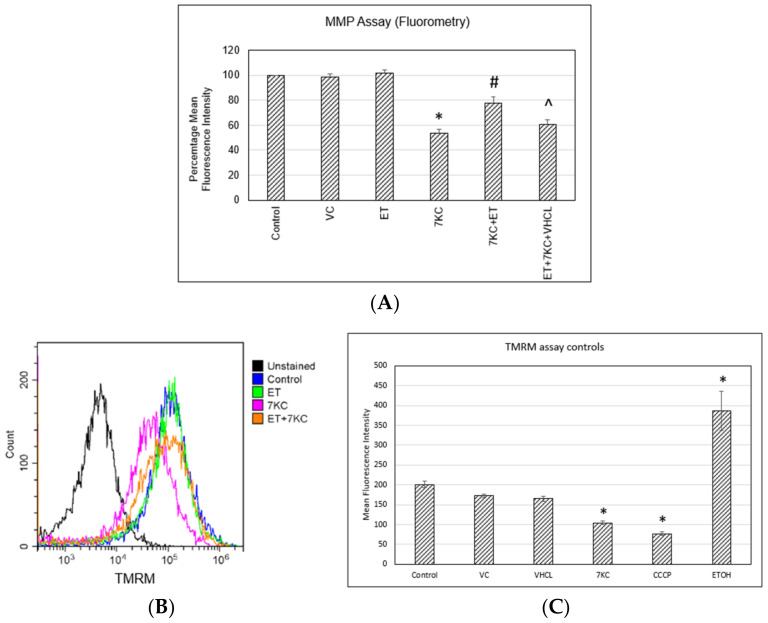
Mitochondrial membrane potential assays. 7KC was found to significantly increase the number of cells with lower mitochondrial membrane potential. ET by itself had no significant effect on mitochondrial membrane potential. However, it significantly reduced the percentage of cells with lower mitochondrial membrane potential when 7KC was added. The effect of 7KC was prevented by ET, and the protective effect was abrogated by coincubation with VHCL. (**A**) The trend was observed in both rhodamine 123 fluorometry and (**B**,**C**) TMRM flow cytometry analysis. (**C**) TMRM assay positive and negative controls using flow cytometry. VC: Vehicle control; ET: 1 mM ET; VHCL: 100 μM VHCL; 7KC: 30 μM 7KC; CCCP: 50 μM CCCP; ETOH: 100 mM. Data are represented as the mean ± SD (n = 6). * Significant difference compared to controls (*p* < 0.05). # Significant difference compared to 7KC. ^ Significant difference compared to ET + 7KC. Data were analysed by one-way ANOVA with Bonferroni’s multiple comparison post-hoc test.

7KC was found to significantly lower mitochondrial membrane potential, as seen with the lower mean fluorescence intensity (MFI) of rhodamine 123 (Figure 5A) and TMRM (Figure 5B) dyes. ET by itself had no significant effect on mitochondrial membrane potential. However, ET significantly restored mitochondrial membrane potential when 7KC was added, which was abrogated by coincubation with VHCL. Carbonyl cyanide 3-chlorophenylhydrazone (CCCP) and ethanol are positive controls for the TMRM assay (MMP levels) (Figure 5C).

### 2.6. ATP Levels

**Figure 6:**
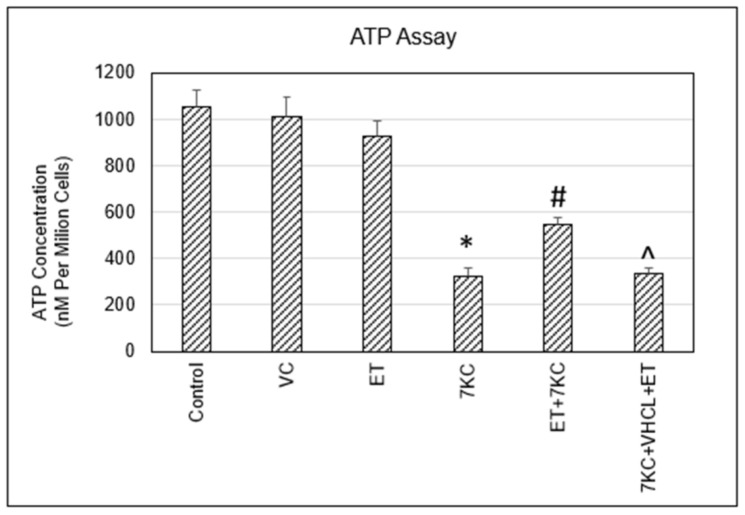
Cellular ATP levels. 7KC treatment resulted in a significant decrease in ATP production, and this decrease was significantly inhibited by ET. Coincubation with VHCL abrogated the protective effect of ET. VC: Vehicle control; ET: 1 mM ET; VHCL: 100 μM VHCL; 7KC: 30 μM 7KC. Data are represented as the mean ± SD (n = 3). * Significant difference compared to controls (*p* < 0.05). # Significant difference compared to 7KC. ^ Significant difference compared to ET + 7KC. Data were analysed by one-way ANOVA with Bonferroni’s multiple comparison post-hoc test.

Quantification of ATP levels could serve as an endpoint functional assay to determine mitochondrial dysfunction. 7KC treatment resulted in a significant decrease in ATP levels. Incubation of cells with ET partially restored ATP levels, which were significantly greater than 7KC-treated cells. Pretreatment with VHCL abrogated the protective effect of ET (Figure 6).

### 2.7. TFAM, NRF-1 and Nrf2 Gene Expression

**Figure 7:**
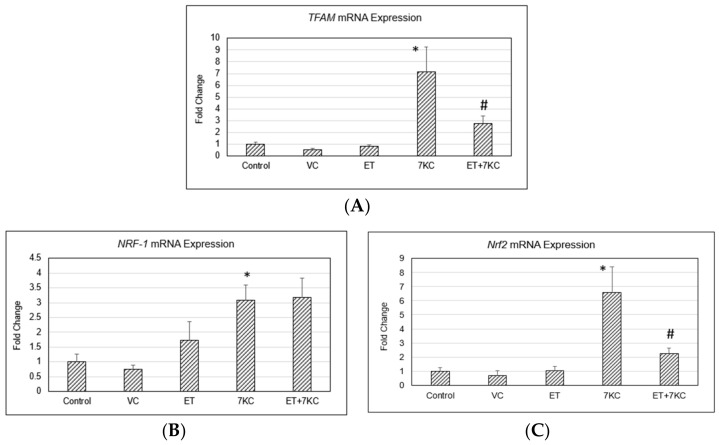
*TFAM*, *NRF-1* and *Nrf2* gene expression. (**A**) *TFAM* mRNA expression was induced by 7KC, but this increase was significantly reduced by ET. As with *TFAM*, there was induction of (**B**) *NRF-1* and (**C**) *Nrf2*, indicating increased overall level of cellular stress after treatment of cells with 7KC, and this increase was similarly significantly decreased by ET. ET did not reduce the 7KC-induced increase in NRF-1. VC: Vehicle control; ET: 1 mM ET; 7KC: 30 μM 7KC. Data are the represented as mean ± SD (n = 4). * Significant difference compared to controls (*p* < 0.05). # Significant difference compared to 7KC. Data were analysed by one-way ANOVA with Bonferroni’s multiple comparison post-hoc test.

Mitochondrial transcription factor A (TFAM) is a nuclear-derived transcription factor involved in mitochondrial DNA maintenance that is inducible by mitochondrial nuclear respiratory factor 1 (NRF-1) transcription factor under stress [28,29]. Nuclear factor erythroid-derived 2-like 2 (Nrf2) is involved in cellular oxidative stress response and various protective mechanisms [30]. *TFAM* mRNA expression was induced by 7KC, but this increase was significantly reduced by ET (Figure 7A). As with *TFAM*, there was induction of *NRF-1* (Figure 7B) and *Nrf2* (Figure 7C), indicating an increased overall level of cellular stress after treatment of cells with 7KC. ET modulated the increased expression of *Nrf2* but not that of *NRF-1*.

### 2.8. Pro-Inflammatory Gene Expression

**Figure 8:**
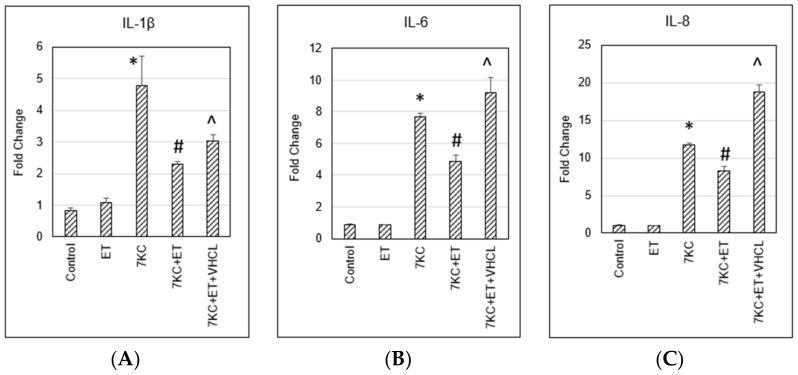
Inflammatory gene expression. 7KC induced increases in (**A**) *IL-1β*, (**B**) *IL-6* and (**C**) *1L-8* and mRNA expression, and these increases were significantly reduced by ET. ET: 1 mM ET; VHCL: 100 μM VHCL; 7KC: 30 μM 7KC. Data are represented as the mean ± SD (n = 4). * Significant difference compared to controls (*p* < 0.05). # Significant difference compared to 7KC. ^ Significant difference compared to ET + 7KC. Data were analysed by one-way ANOVA with Bonferroni’s multiple comparison post-hoc test.

7KC induced a significant increase in *IL-1β*, *IL-6* and *1L-8* mRNA expression, and these increases were significantly reduced by ET (Figure 8).

## 3. Discussion

The present study aimed to elucidate the effect of ET on 7KC-induced damage in hCMEC/D3 brain endothelial cells. The hCMEC/D3 model is suitable for neurovascular and neurodegenerative disease studies because the cell line stably maintains a BBB phenotype [31], and BBB dysfunction is now well recognised to be associated with various neurodegenerative diseases [32]. Studies using this cellular model in Alzheimer’s disease [33,34] and Parkinson’s disease [35,36] have provided crucial insights on disease pathology and possible treatments. 7KC was found to induce approximately 60% cell death and loss of cell viability in hCMEC/D3 brain endothelial cells, using Trypan blue and MTS assay, respectively, and 7KC-induced cytotoxicity was significantly reduced by cotreatment with ET. The protective effect of ET was abolished when cells were treated with a nonspecific inhibitor of the ET transporter OCTN1, VHCL, together with ET. These results suggested that the protective effect of ET was dependent on cellular uptake of ET and not through, perhaps, extracellular neutralisation of 7KC. Moreover, our LC-MS results confirmed that VHCL decreased ET uptake into cells. To confirm that OCTN1 is present in brain endothelial cells, RT-PCR was performed on these cells. The results showed that VHCL or addition of ET did not alter the expression of *OCTN1*. In contrast, 7KC induced a significant, 4-fold increase in *OCTN1* mRNA expression. This increase was not affected by the addition of ET or VHCL. The findings demonstrate upregulation of *OCTN1* in the brain endothelial cells upon 7KC-induced injury. This finding could perhaps be a protective mechanism to increase ET uptake in injured tissues, as previously suggested [37,38,39]. Previous studies have also found that OCTN1 is important in the cellular uptake of ET in endothelial cells [22] and that OCTN1 levels can be upregulated in response to tissue injury, e.g., in fatty liver [40] or kidney disease [41].

One of the known effects of 7KC on cells is the disruption of calcium homeostasis, thereby causing an increase in intracellular free calcium levels. Excessive increases in cytosolic calcium concentration could trigger pathways leading to apoptosis [42,43,44]. Influx of calcium has been found in endothelial cells after treatment with 7β-hydroxycholesterol, cholesterol 5β, 6β-epoxide, cholesterol 5α, 6α-epoxide and 7KC [45]. The influx of calcium could occur via specific calcium channels on the cells. A calcium channel blocker, azelnidipine, reduced RelA (p65) nuclear translocation in 7KC treated human aortic endothelial cells [46]. In addition, 7KC-induced increases in intracellular free calcium could occur through release from intracellular stores [14]. We confirmed that 7KC treatment increased intracellular free calcium and that the increase was significantly modulated by ET. This effect could be due to the ability of ET to reduce oxidative stress [47] and/or chelate divalent metal ions [48,49,50], while the increase in chelation could increase sequestration of cytosolic calcium ions. Verapamil alone does not appear to affect intracellular free calcium levels [51], but coincubation with VHCL abrogated the ET-induced reduction in intracellular free calcium levels, indicating that modulation is likely to occur intracellularly, e.g., via modulation of intracellular stores in the endoplasmic reticulum or mitochondria, and is not due to external chelation of calcium by ET. Together, our results indicate that ET is effective in preventing 7KC-induced increases in intracellular free calcium.

7KC appeared to induce an increase in ROS production in brain endothelial cells, as shown by DCFDA assay, although the limitations of this assay are well known [27]. This outcome is very similar to the results of another study, which showed ROS/RNS production and mitochondrial DNA damage after 7KC treatment in human retinal pigment epithelial cells in vitro [52]. Addition of ET significantly reduced ROS production, and such protection was abrogated by VHCL. We validated the DCFDA results with MitoSOX assay, which appears to detect mitochondrial ROS production, and we confirmed that the 7KC-induced increase in mitochondrial ROS was significantly reduced by ET. These results highlight the importance of mitochondria in ROS formation induced by 7KC and the ability of ET to reduce such ROS production.

Increased intracellular free calcium levels could trigger increased calcium buffering from mitochondria, contributing to mitochondrial calcium overload and a resultant loss of mitochondrial membrane potential (MMP) [16]. In this study, we demonstrated 7KC-induced damage to mitochondria, as determined by MMP assays. The deleterious effects of 7KC on MMP were ameliorated by ET, and this protective effect was abrogated by coincubation with VHCL. Loss of mitochondrial membrane potential could result in ROS production [53] and account for the increase in mitochondrial ROS production, as shown by MitoSOX assay above. The ability of 7KC to disrupt mitochondrial membrane potential has also been reported in human retinal pigment epithelial cells [52], 158N murine oligodendrocytes [54] and N2a mouse neuronal cells [18].

Damage to mitochondria could lead to a reduction in energy production critical for cellular metabolic processes. Defective energy metabolism has an important role in aging and neurodegenerative diseases [55,56,57]. In this study, we showed that ET was able to significantly inhibit 7KC-induced decreases in ATP levels in brain endothelial cells, and coincubation with VHCL abrogated the protective effect of ET, consistent with an intracellular effect of ET. 7KC-induced loss of mitochondrial membrane potential and ATP production have previously been shown in human aortic smooth muscle cells [58] and mouse endothelial cells [59]. In addition to oxidative phosphorylation, 7KC-induced loss of cellular ATP could be due to perturbations of the glycolysis pathway [60].

The decrease in mitochondrial stress induced by ET is evidenced by a drop in *TFAM* gene expression. TFAM is a downstream activation product of NRF-1 activation, and both NRF-1 and TFAM play key roles in regulation of oxidative stress and maintenance of mitochondrial DNA [28,29]. 7KC induced an increase in *NRF-1* and *TFAM* expression, suggesting mitochondrial stress. As with *NRF-1* and *TFAM*, there was induction of *Nrf2*, indicating an increased overall level of cellular stress [28]. An oxidative stress-induced increase in mitochondrial biogenesis genes was reported in HeLa cells [61]. The contribution of TFAM to intracellular calcium homeostasis has also been discussed [62].

Damage to mitochondria could also lead to activation of the inflammasome [63] and induction of inflammation [64]. Activation of NLRP3 inflammasome has been found in endothelial cells after treatment with 7KC [65,66,67], retinal pigment epithelial cells, and bone marrow-derived cells, including microglia [66]. In this study, we showed significant increases in the mRNA expression of the inflammatory cytokines *IL-1β*, *IL-6* and *IL-8* after 7KC treatment, and these increases were modulated by ET. The results are consistent with our previous findings that there are increases in *NF-κB*, *IL-1β*, *IL-6*, and *TNF-α* expression and COX-2 enzymatic activity after 7KC treatment of endothelial cells, and such increases are ameliorated by ET [26].

## 4. Materials and Methods

### 4.1. Chemicals

7-ketocholesterol was purchased from Cayman Chemical (Ann Arbor, MI, USA). L-ergothioneine and L-ergothioneine-d_9_ (ET-d_9_; deuterated internal standard) were kindly provided by Tetrahedron (Paris, France). Tert-butyl hydroperoxide (TBHP), carbonyl cyanide 3-chlorophenylhydrazone (CCCP) and verapamil hydrochloride (VHCL), a nonspecific OCTN1 inhibitor, was purchased from Sigma Aldrich (St. Louis, MO, USA). Cells were cultured in Endothelial Basal Medium (EBM-2) (Lonza, Bend, OR, USA) with HyClone antimycotic/antibiotic solution (1×, Thermo Fisher Scientific, Waltham, MA, USA) and growth factors from the EGM-2MV kit (Lonza). The EGM-2MV kit contains gentamicin/amphotericin-B (GA), human epidermal growth factor (hEGF), ascorbic acid, vascular endothelial growth factor (VEGF), R3-insulin-like growth factor-1 (R3-IGF-1), hydrocortisone, human fibroblast growth factor-beta (hFGF-β) and foetal bovine serum (5% *v/v*).

### 4.2. Cell Culture

Human brain endothelial cells (hCMEC/D3), derived from microvessels in the human temporal lobe, were purchased from EMD Millipore (Temecula, CA, USA) and cultured according to Weksler et al. [68]. hCMEC/D3 cells between passage 6 and 20, were utilised for all experiments. Dose response assays were performed, and 30 μM 7KC, 1 mM ET and 100 μM VHCL were determined to be the optimal concentrations for toxicity, protection, and inhibition, respectively, similar to prior in vitro studies [22,26,69,70]. In all cases, cells were preincubated with 1 mM ET for 1 h prior to coincubation with 7KC for 24 h. If VHCL was added, a 2-h preincubation time was allocated prior to addition of ET.

### 4.3. Trypan Blue Cell Viability Assay

Trypan blue assay, which utilises an exclusion method, was performed to analyse the effects of 7KC and ET on the viability of cells, whereby nonviable cells take up the dye, but viable cells do not. Treated cells were detached with trypsin (0.25%) (Omega Scientific, Los Angeles, CA, USA) for 3 min before centrifugation for 5 min at 1000× *g*. The supernatant was discarded, and the pellet resuspended in fresh EBM-2 media, and a portion of the cell suspension was mixed 1:1 with trypan blue (0.4%) (Thermo Fisher Scientific) and loaded into cell counting chambers for analysis by an automated cell counter (TC10, Bio-Rad, Hercules, CA, USA).

### 4.4. MTS Assay

MTS cell viability assay is based on the principle of MTS tetrazolium compound reduction by viable cells to generate coloured formazan dye, which can be measured by absorbance at 490 nm. Twenty microlitres of CellTiter Aqueous One Solution (MTS proliferation assay, Promega, Madison, WI, USA) were added to treated cells on a 96 well plate. Subsequently, a Synergy H1 Microplate Reader (BioTek, Winooski, VT, USA) was utilised to read the absorbance at 490 nm.

### 4.5. Cellular ET Uptake and Liquid Chromatography Mass Spectrometry

Cells were grown to 80% confluence and treated prior to ET extraction from cells. Samples were washed thrice with ice cold PBS before addition of methanol spiked with internal standard (ISTD) comprising of ET-d9. Next, samples were centrifuged at 20,000× *g* at 4 °C for 10 min, and the supernatant was collected in glass vials before being evaporated at 37 °C under an N_2_ stream. The contents of the glass vials were resuspended in methanol solution, and ET levels were analysed via liquid chromatography mass spectrometry (LC-MS/MS) using an Agilent 1290 UPLC system coupled with an Agilent 6460 ESI mass spectrometer (Agilent Technologies, Santa Clara, CA, USA). Samples were kept at 10 °C in the autosampler. Two microlitres of the processed samples were injected into a Cogent Diamond-Hydride column (4 µm, 150 × 2.1 mm, 100 Å; MicroSolv Technology Corporation, Brunswick, NC, USA) maintained at 30 °C. Solvent A was acetonitrile in 0.1% formic acid, and Solvent B was 0.1% formic acid in ultrapure water. Chromatography was performed at a flow rate of 0.5 mL/min using the following gradient: 1 min of 20% Solvent B, followed by a 3-min gradient increase in Solvent B to 40% to elute ET. The retention time for ET is 4.2 min.

Mass spectrometry was performed using the positive ion, electrospray ionisation mode, with multiple reaction monitoring (MRM) for quantification of specific target ions. The capillary voltage was set at 3200 V, and the gas temperature was kept at 350 °C. The nitrogen sheath gas pressure for nebulising samples was at 50 psi, and the gas flow was set at 12 L/min. Ultra-high purity nitrogen was used as collision gas. The precursor to product ion transitions and fragmentor voltages (V)/collision energies (eV) for each compound were as follows: ET; 230.1 → 186, 103 V/9 eV and ET-d9; 239.1 → 195.1, 98 V/9 eV.

### 4.6. Measurement of Intracellular Free Calcium, Mitochondrial Membrane Potential, and Cellular/Mitochondrial Reactive Oxygen Species (ROS)

Flow cytometry and fluorometry were employed to assess mitochondrial membrane potential and ROS production. Cells were first detached, and cell pellets were washed twice with PBS. Cells were incubated for 30 min with their respective stains in the absence of light before conducting flow cytometry analysis. Intracellular free calcium was quantified using Fluo-4 (Thermo Fisher Scientific). Mitochondrial membrane potential (MMP) was quantified using rhodamine 123 (Thermo Fisher Scientific) and tetramethyl rhodamine methyl ester perchlorate (TMRM) (Sigma Aldrich). For the controls, pretreatment with 100 mM ethanol (ETOH) and 50 μM CCCP for 30 min was used to induce hyperpolarisation and depolarisation of mitochondrial membrane potentials, respectively. Cellular ROS production was quantified using cell-permeant 2′,7′-dichlorodihydrofluorescein diacetate (H_2_DCFDA) (Thermo Fisher Scientific) and mitochondrial ROS (mROS) production was quantified using MitoSOX assay (Thermo Fisher Scientific). Tert-butyl hydroperoxide (TBHP) at a concentration of 50 μM was used as a positive control to induce oxidative stress.

Flow cytometry measurements were performed using a Cytoflex LX flow cytometer (Beckman Coulter Life Sciences, Brea, CA, USA), using 10^6^ cells per sample for analysis with 10,000 events per sample recorded. The FL1 channel was used to quantify intracellular free calcium, MMP, ROS, mROS (Fluo-4, Ex/Em = 488/525 nm; rhodamine 123, Ex/Em = 507/529 nm; TMRM, Ex/Em = 555/575 nm; H_2_DCFDA, Ex/Em = 495/527 nm; MitoSOX, Ex/Em = 510/580 nm). Fluorometry measurements were performed using a Synergy H1 Microplate Reader (BioTek). For flow cytometry, fluorescent signals were measured on a logarithmic scale of four decades of log. Raw data were processed using FlowJo software, version 10.5.3.

### 4.7. ATP Assay

ATP levels in cells were quantified using a commercial ATP determination kit (Thermo Fisher Scientific). This bioluminescence assay is based on the reaction of ATP with recombinant firefly luciferase and its substrate luciferin. Cells were collected via trypsinisation and quantified by Trypan blue assay to ensure all samples contained equal numbers of cells (10^6^ cells). Upon centrifugation and removal of supernatant, 1 mL of boiling ultrapure water from an Arium pro^®^ ultrapure system was added into the cell pellet and incubated in a water bath for 10 min at 100 °C [71]. Samples were then cooled on ice for 30 s, and the supernatant was utilised for ATP assay per the manufacturer’s instructions. Luminescence readings of samples were performed using a Synergy H1 Microplate Reader (BioTek) alongside ATP standards.

### 4.8. Quantitative RT-PCR

TRIzol Reagent (Thermo Fisher Scientific) was used for RNA extraction per the manufacturer’s instructions. cDNA was produced from reverse transcription of 1000 ng of RNA (High-Capacity cDNA Reverse Transcription Kit; Applied Biosystems, Waltham, MA, USA) using a T-Personal Thermocycler (Biometra, Gottingen, Germany) programmed at 25 °C for 10 min and 37 °C for 30 min, followed by 85 °C for 5 min. qPCR was performed to quantify *OCTN1*, *NRF-1*, *Nrf2*, *TFAM*, *IL-6*, *IL-8*, and *IL-1β* mRNA expression (*OCTN1*: Hs.310591_m1; *NRF-1*: Hs.654363_m1; *Nrf2* or *NFE2L2*: Hs.744006_m1; *TFAM*: Hs.594250_m1; *IL-1β*: Hs.1555410_m1; *IL-6*: Hs.174131_m1; *IL-8*: Hs.174103_m1), using TaqMan Gene Expression Assay Probes (Applied Biosystems) and TaqMan Universal PCR Master Mix (Applied Biosystems). GAPDH (Hs99999903_m1). The qPCR condition was performed using a 7500 Real-time PCR System (Applied Biosystems) with conditions of 95 °C for 10 min, followed by 95 °C for 15 s and 60 °C for 1 min, for 40 cycles. Subsequently, the relative mRNA expressions for the respective genes of interest were quantitated via comparative CT (ΔΔCT) method.

### 4.9. Statistical Analysis

All data are presented as the mean ± standard deviation (SD). Comparisons in the Trypan blue, MTS, gene expression, flow cytometry, and fluorometry assays were performed by one-way ANOVA with Bonferroni’s post-hoc correction, using GraphPad Prism software, version 9. A *p*-value < 0.05 was considered significant.

## 5. Conclusions

The above studies showed a cytoprotective effect of ET on 7KC-induced toxicity in human brain endothelial cells. Our data suggest that the protective effect of ET on 7KC-induced injury was, at least in part, mediated by prevention of mitochondrial dysfunction as seen through a reduction in mitochondrial membrane damage, a decrease in ROS, and an increase in ATP levels. These results add to our previous findings that 7KC has cytotoxic and proinflammatory effects on brain endothelial cells, which are protected by ET. It is hoped that these results shed light on the role of ET in the prevention and treatment of neurovascular brain disorders, in which we recently showed a neuroprotective effect of ET in rodent models of stroke [72]. Further studies are needed to elucidate the protective effect of ET against 7KC in vivo.

## Data Availability

The data are available upon reasonable request.
